# Optimized Culture Conditions for Improved Growth and Functional Differentiation of Mouse and Human Colon Organoids

**DOI:** 10.3389/fimmu.2020.547102

**Published:** 2021-02-12

**Authors:** Sarah S. Wilson, Martha Mayo, Terry Melim, Heather Knight, Lori Patnaude, Xiaoming Wu, Lucy Phillips, Susan Westmoreland, Robert Dunstan, Edda Fiebiger, Sonia Terrillon

**Affiliations:** ^1^ Foundational Immunology, AbbVie, Cambridge Research Center, Cambridge, MA, United States; ^2^ Immunology Pharmacology, AbbVie, AbbVie Bioresearch Center, Worcester, MA, United States

**Keywords:** intestinal organoids, enteroids, colonoids, Wnt3 signaling, Goblet cells, adult stem cells, intestinal epithelial cells, differentiation

## Abstract

**Background & Aims:**

Diligent side-by-side comparisons of how different methodologies affect growth efficiency and quality of intestinal colonoids have not been performed leaving a gap in our current knowledge. Here, we summarize our efforts to optimize culture conditions for improved growth and functional differentiation of mouse and human colon organoids.

**Methods:**

Mouse and human colon organoids were grown in four different media. Media-dependent long-term growth was measured by quantifying surviving organoids *via* imaging and a cell viability readout over five passages. The impact of diverse media on differentiation was assessed by quantifying the number of epithelial cell types using markers for enterocytes, stem cells, Goblet cells, and enteroendocrine cells by qPCR and histology upon removal of growth factors.

**Results:**

In contrast to Wnt3a-conditioned media, media supplemented with recombinant Wnt3a alone did not support long-term survival of human or mouse colon organoids. Mechanistically, this observation can be attributed to the fact that recombinant Wnt3a did not support stem cell survival or proliferation as demonstrated by decreased LGR5 and Ki67 expression. When monitoring expression of markers for epithelial cell types, the highest level of organoid differentiation was observed after combined removal of Wnt3a, Noggin, and R-spondin from Wnta3a-conditioned media cultures.

**Conclusion:**

Our study defined Wnt3a-containing conditioned media as optimal for growth and survival of human and mouse organoids. Furthermore, we established that the combined removal of Wnt3a, Noggin, and R-spondin results in optimal differentiation. This study provides a step forward in optimizing conditions for intestinal organoid growth to improve standardization and reproducibility of this model platform.

## Introduction

The intestinal epithelium forms the largest of the body’s mucosal surfaces with a single layer of polarized cells covering a surface area of ~400 m^2^. In addition to their role in digestion and water/nutrient resorption, intestinal epithelial cells (IECs) function as a physical and biochemical barrier to separate host tissue from commensal bacteria and digestion end products in the intestinal lumen (1). The latter properties of the intestinal epithelium are a key factor for controlling the maintenance of intestinal homeostasis, host pathogen interactions, and mucosal inflammation. Over the last three decades, a rapid increase in intestinal disorders, such as inflammatory bowel diseases (IBD) or food allergy, has been observed in industrialized countries (2–4). Commonalities in the pathophysiology of these disorders imply that the ability of the mucosal barrier to maintain integrity and mount tolerance to commensals bacteria and food products while remaining responsive to pathogens is challenged (5, 6).

A deeper understanding of the cellular and molecular mechanisms that control IEC functions in health and disease requires access to defined *in vitro* culture systems. Immortalized intestinal epithelial cells of human and murine origin have been available for research purposes for decades. The most common models rely on the use of colonic adenocarcinoma cell lines which retain altered cellular pathways of transformed cells. Such cell cultures, particularly in their polarized form, recapitulate some features of the intestinal epithelium and are useful for studying functions such as apical and basolateral distribution of proteins of research interest, para- and trans-cellular transport mechanisms, or the formation of tight junctions (7). However, these cultures cannot recapitulate the subcellular composition of the intestinal epithelium as found *in vivo* and are not useful for studying host diversity. Therefore, experimental observations with cell line model systems, while providing powerful insights into molecular mechanisms of polarized cell layers, are hard to interpret with regards to their relevance in the physiological setting of health and/or disease.

Advances in our understanding of adult stem cells and the characterization of the adult intestinal niche allowed for the generation of intestinal organoids and closed the significant gap in our experimental tool box for studying IECs in functionally relevant settings (8, 9). One common method for the generation of intestinal organoids is based on the use of tissue-derived stem cells isolated from human biopsies or surgical specimens, which are differentiated into “epithelial only” cultures commonly referred to as enteroids or colonoids dependent on the source of intestinal tissue the stem cells are derived from (i.e., small bowel vs. colon) (10, 11). An alternative method uses pluripotent stem cells, of embryonic origin or from reprogrammed somatic cells, and gives rise to so called “organoids” that contain intestinal epithelial cells and stromal mesenchyme (12). In both systems, stem cells produce self-organizing cultures that contain multiple differentiated intestinal epithelial cell types including enterocytes, Goblet, Paneth, and enteroendocrine cells. Because of our interest in using these cultures for the development and assessment of curative or preventive therapies for intestinal inflammatory diseases, we chose to focus on studying colonoids from mouse and human tissue in this study.

Common consensus has been established that successful colonoid cultures rely on the maintenance and propagation of intestinal stem cells which is dependent on growth factors in culture medium (13). A source of EGF or an activator of the EGFR pathway and downstream ERK transcription contribute to the maintenance of stemness, as does Notch signaling provided by niche resident neighboring cells to stem cells. Bone-morphogenic protein signaling inhibits stemness and, therefore, the addition of noggin or other proteins that block this signaling axis is necessary. Finally, most critical to the maintenance of intestinal stem cells is the activation of canonical Wnt signaling. This is provided by the addition of both canonical Wnt proteins, such as Wnt3a, as well as the Wnt signaling potentiator, R-spondin, to the media.

Initial reports describing intestinal organoid cultures relied on media that included commercially available recombinant growth factors, EGF, Noggin, Wnt3a and R-spondin, as well as additional additives based on previous work in stem cell systems (10, 11). Although successful as demonstrated in many publications, the reliance of culture media on purified proteins is both expensive and creates difficulties for scaling and reproducibility due to the necessity of making up culture media with many components fresh each week. Subsequently, research attempts focused on establishing strategies to streamline and reduce media cost by utilizing conditioned media as a source of some or all the growth factors and removing many of the culture media additives (14, 15). These efforts culminated in a publication in 2015, describing the growth of human and mouse organoids in conditioned media derived from an R-spondin-Noggin-Wnt3a producing cell line (14, 16). To date, numerous methods and culture conditions have been described to support the long-term growth of colonoids. However, no published studies that directly compare culture approaches are available to the research community yet leaving a gap in our knowledge.

A detailed comparative evaluation of proliferative features of the stem cell compartment under different culture conditions was one purpose of this study. Importantly, maintenance of stemness and differentiation status of enteroid and organoid cultures are inversely corelated and a switch from propagation to differentiation culture conditions is required for establishing models that contain all epithelial cell types of the epithelial barrier *in vivo*. Another goal was, thus, to perform a systematic analysis of how a switch from propagation to differentiation medium affects epithelial cell type composition across species. These are immanent questions to address because the debate of how and if organoid-derived epithelial cell types recapitulate their *in vivo* counterparts and composition is still ongoing amongst experts in the field (17, 18).

We performed a detailed comparison of effects of different media conditions on the long-term growth and functional differentiation of human and mouse colonoids. Our results support the use of conditioned-media-derived Wnt3a for reproducible long-term mouse and human colonoid culture. Furthermore, we demonstrated that differentiation conditions are not identical across species and that removal of different growth factors from propagation cultures results in distinct epithelial phenotypes with species dependent differences. Comparisons of the epithelial cell composition in our colonoid cultures to that observed histologically in the colon of naïve mice demonstrated that our culture systems are modeling the mid colon as compared to the distal colon. The results provide a methodological framework and experimental rational for the development of colonoid models that more faithfully recapitulate the multi-dimensional microenvironment of the intestinal epithelium. In conclusion, this study provides a step forward in the development of improved cellular models of the intestinal epithelium that will facilitate mechanistic exploration of disease-associated pathophysiologic mechanisms.

## Materials and Methods

### Ethics Statement.

*Animal Procedures:* Experiments that involved live animals (specifically, collection of colon tissue for the generation of colon stem cell derived colonoids and use of naïve C57BL/6 mice for histological evaluation) were approved by the Institutional Animal Care and Use Committee of AbbVie.

*Human Tissue:* Human samples for organoid lines were obtained from the Stappenbeck and Ciorba labs at the University of Washington in St. Louis as previously described (14). Formalin-fixed paraffin embedded human colon tissue for histological evaluation was obtained from the University of Massachusetts Medical School, Worcester, Massachusetts. The study was approved by the Institutional Review Board of University of Massachusetts School of Medicine. Written informed consent was obtained from all donors. For this study, tissue was from a non-IBD patient undergoing surgical resection and tissue used was the normal adjacent tissue to the abnormal resection.

### Colonoid Establishment

For mouse colonoids, crypts were isolated using EDTA chelation from the colons of C57Bl/6 mice purchased from Jackson laboratories (Bar Harbor, Maine) as previously described (11, 19). Briefly, mice were sacrificed using CO_2_ and the colon was removed by cutting the proximal colon from the cecum and the distal colon at the anal margin. After flushing 2–3 times with dPBS [w/o Ca2+ and Mg2+] using a gavage needle on a 10 cc syringe, the colon was opened longitudinally and swirled in cold dPBS in a petri dish to rinse. The debris and the mucosa removed by scraping with a glass coverslip and then tissue was minced into 2 mm fragments in PBS, pipetted up and down three times with a 10 ml pipette, and then new dPBS was added. This was repeated 5–10 times until the supernatant was clear. For crypt isolation, tissue fragments were transferred to tubes pre-coated with 1% BSA, and incubated in chelating buffer [PBS w/o Ca and MG (Sigma # D8537-500ML), 2 mM EDTA (ThermoFisher Scientific # AM9260G), 2 mM D-sorbitol (Sigma #S1876), 43.4 mM Sucrose powder (Sigma # S1888)] for 30 min at 4°C on a rocking platform. After incubation, supernatant was discarded, and the tissue was resuspended in fresh chelating buffer by pipetting up and down 3 times followed by vigorous shaking for 60 s. This filtrate is collected and labeled as fraction 1, and subsequent fractions are collected as needed (until crypts are no longer present, generally 5–7 fractions) following the above protocol. The quality of each fraction was assessed under a microscope and fractions enriched for intestinal crypts were selected, pooled, filtered through a 70 um cell strainer and centrifuged (4°C, 5 min, 600 g). After spinning, the supernatant was discarded and the pellet was resuspend in 10 ml washing medium [Advanced DMEM/F12 (ThermoFisher Scientific #12634), 1x GlutaMAX (ThermoFisher Scientific #35050), 1x Pen/Step (ThermoFisher Scientific #15140), 1x Hepes (ThermoFisher Scientific #15630)]. For plating, appropriate volume of crypt fraction (500 intact epithelial units per well) was pelleted and resuspended in Growth Factor reduced Phenol Red-free Matrigel (Corning BD #356231). Fifty ul of cell-Matrigel suspension was plated in the center of each well of a 24-well plate. Matrigel polymerized for 15 min and was overlaid with 500 ul of growth media per well.

For the comparative studies with human lines, previously established human colonoids derived from rectal biopsy samples from healthy donors were obtained from the laboratories of Drs. Stappenbeck and Ciorba at the University of Washington in St. Louis (14).

### Production of Wnt3a Conditioned Media and Wnt3a-Noggin-Rspondin Conditioned Media

The L-Wnt3A cell line was purchased from ATCC (ATCC^®^ CRL-2647™) and Wnt3a conditioned media was produced following the ATCC recommendations. Briefly, L-Wnt3A cells were thawed into one 10 cm^2^ dish and grown in L-cell media [DMEM high glucose (Sigma #D6429), 100X Penicillin/Streptomycin (ThermoFisher Scientific #15140), 10% FBS (ThermoFisher Scientific #10438026)] for 2–3 days, before passaging into two 150 cm^2^ flasks with L-cell media + G418 at 0.4 mg/ml (ThermoFisher Scientific #10131035). Cells were then split to ten 150 cm^2^ flasks and placed in primary culture media [Advanced DMEM/F12 (ThermoFisher Scientific #12634-010), 1x GlutaMAX (ThermoFisher Scientific #35050), 1x Penicillin/Streptomycin (ThermoFisher Scientific #15140) and 20% FBS (Sigma #F2442)]. After 4 days conditioned media was collected in 50 ml tubes, spun down at 3,000 RPM, RT, for 5 min, and then supernatant filtered over 0.22 uM PES low protein binding filter before storage at 4C. Media was replaced with fresh primary culture media and after 3 additional days the day 5–7 conditioned media was collected and processed as above. Day 1–4 conditioned media was combined with day 5–7, aliquoted, and stored at -20°C.

L-WRN cell line (ATCC# CRL-3276™) was kindly provided by the Ciorba and Stappenbeck labs at Washington University in St. Louis. In order to minimize passaging of the L-cells for conditioned media production, the initial L-WRN cell vial was thawed and plated into one 150 cm^2^ flask with 25 ml L-cell media. Cells were grown 1–2 days until confluent and then passaged to eight 150 cm^2^ flasks with 0.5 mg/ml G418 and 0.5 mg/ml Hygromycin B (ThermoFisher Scientific #10687010). Cells were frozen (8 vials/flask, 64 vials total) once they became confluent and a fresh vial of L-WRN cells was thawed for every batch of L-WRN conditioned media produced. For conditioned media production, cells were thawed on a Monday and transferred to a 75 cm^2^ flask with 15 ml L-cell media. Cells were grown until they became confluent (2 days) and passaged to one 150 cm^2^ flask under 500 ug/ml G418 and 500 ug/ml Hygromycin B selection. After 2 days of growth, cells were passaged to ten 150 cm^2^ flasks and maintained under selection. After 3 days of growth the cells become overconfluent and media becomes a used orange color, at which point the cells were washed twice with 20 ml of PBS per flask, once with 10 ml of primary culture media per flask, before addition of 25 ml of primary culture media per flask and incubation for 24 h. Conditioned media was collected into 50 ml tubes, centrifuged at 3,000 rpm for 5 min and supernatant transferred to a sterile 2 l bottle and stored at 4°C. Fresh media was added to flasks and conditioned media collected from the 2nd, 3^rd^, and 4th days in the same 2 l bottle. Conditioned media was filtered using a 0.22 uM PES filter, and aliquots frozen at -80°C until needed. For coloniod growth, conditioned media is diluted with Primary culture media to 50%. A note on FBS: In our hands, differences in the content and quality of FBS significantly impacted the activity of the conditioned media. We recommend using Sigma FBS catalogue #F2442 or asking your vendor to provide you with their lot of FBS that most closely matches these specifications. Before use, FBS was heat inactivated by incubation in a 56°C water bath, followed by immediate chilling in an ice water slurry. After heat-inactivation, FBS was aliquoted and stored at -80°C.

### Routine Colonoid Culture and Passaging

Mouse and human colonoids were routinely passaged following the protocol with slight modifications (20). For passaging, media was aspirated from each well and 0.5 ml of PBS was added to each well, followed by scraping with the tip of a P1000 pipette to break up the Matrigel plug. The volume was then transferred to a 15 ml conical tube (up to 8–10 wells per tube), wells were washed with another 0.5 ml PBS-EDTA [PBS without Calcium and Magnesium (Sigma #D8537), 2 mM EDTA solution (ThermoFisher Scientific #15575-038)] and this volume was added to the previous volume. Samples were spun at 600 g, RT for 5 min, and then supernatants were aspirated, leaving the Matrigel gel plug containing colonoids behind. Two hundred ul of room temperature trypsin-EDTA (10x Trypsin-EDTA (Sigma #T4549) diluted to 1x in PBS-EDTA solution above) was added to each tube, mixed by pipetting up and down, and then placed in a 37°C water bath for 60 s for mouse colonoids and 120 s for human colonoids. Trypsin was quenched by the additions of 1 ml washing media (Advanced DMEM/F12, 1x GlutaMAX, 1x Pen/Step, 10% FBS (Sigma #F2442) to each tube, followed by vigorous pipetting to dissociate spheroids using a P1000 at full volume (pipette 60–80 times per sample, alternating between slow up and down and quick, fast strokes with 0.25–0.5 ml total.). Successful dissociation at this point will yield single cells. After dissociation, an additional 4 ml of washing media was added to the tubes and they were spun down at 600 g, RT for 5 min. Pellets were resuspended in 1 ml washing media and transferred to a 1.5 ml tube. Colonoids were again pelleted by spinning at 600 g for 5 min, RT where the 1.5 ml tubes were nested in open top 50 ml conical vials. After aspiration, 1.5 ml tubes were placed on ice and appropriate volumes of Matrigel was added as outlined below.

**Figure 1:**
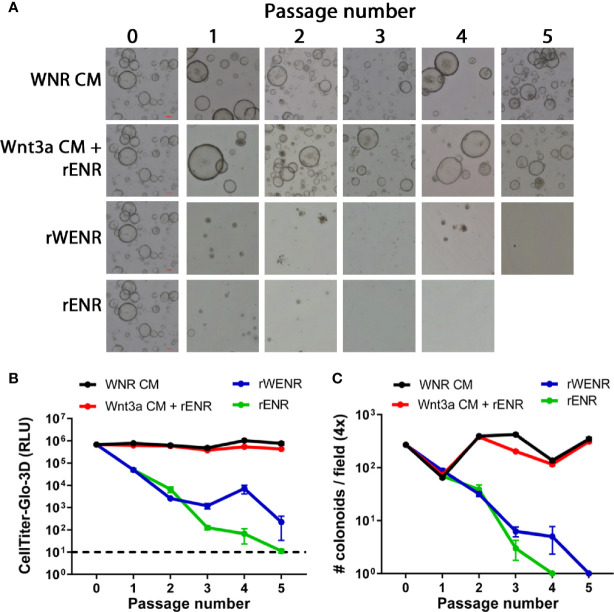
Side-by-side culture comparisons demonstrate a crucial role of Wnt3a-conditioned media for the long-term survival of mouse colonoids. Survival of mouse colonoids derived from the crypts of C57Bl/6 mice was compared in different culture conditions over five passages. Media culture conditions from the top: Wnt3a-Noggin-Rspondin conditioned media (WNR CM), Wnt3a-conditioned media supplemented with recombinant EGF, Noggin and R-spondin (Wnt3a CM + rENR), media with recombinant Wnt3a, EGF, Noggin, and R-spondin (rWENR), and media with recombinant EGF, Noggin, and R-spondin but no recombinant Wnt3a (rENR). **(A)** Representative 4x bright field images for each growth condition medium at every passage. Scale bars indicate 100 microns. **(B, C)** Colonoid-forming efficiency was assessed before each passage using **(B)** the Cell Titer-Glo 3D luminescent cell viability assay or **(C)** counting the number of colonoids per visual field. In B, 3 wells of colonoids were quantified at every passage and in A, images were taken and quantified from 4 wells of colonoids at every passage. Error bars represent the average of the 3 or 4 wells, and representative images and survival from two independent experiments is shown.

*Consistent Split to Compare Growth* (**Figures 1**, **4**). Colonoids were split at a ratio of 1:4. Specifically, one wells of colonoids were split and resuspended in Matrigel equivalent for four wells of colonoids. Subsequent passages followed the same split paradigm.

**Figure 2:**
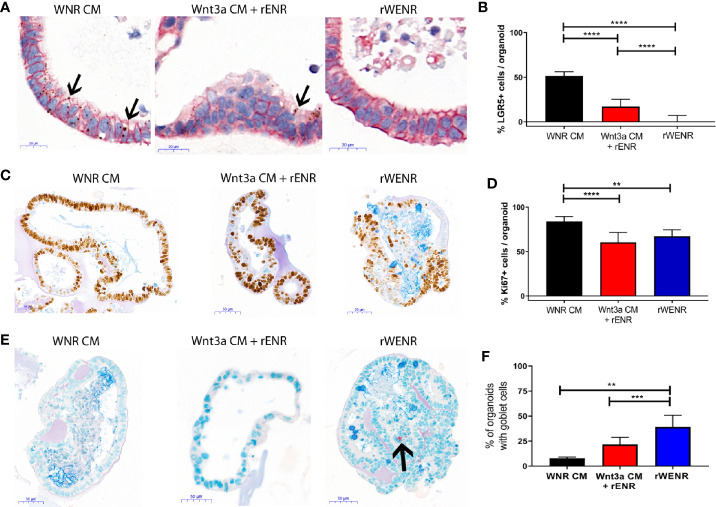
Comparative analysis of culture conditions shows that recombinant Wnt3a does not support maintenance and propagation of stem cells in mouse colonoids. Mouse colonoids derived from the crypts of C57Bl/6 mice were cultured for six days in WNR CM, Wnt3a CM + rENR, or rWENR medium and analyzed for phenotypic alterations, proliferation, goblet cell numbers, and Wnt signaling activity using the following readouts: **(A, B)** Colonoids were stained with *in situ* hybridization for LGR5 for the quantification of stem cells (brown dots), immunohistochemistry for EpCam to stain all epithelial cells, and nuclei stained with hematoxylin; Arrows indicate LGR5 positive areas **(C, D)** immunohistochemistry and quantification of Ki67 as a proliferation marker, with an Alcian Blue counterstain (mucus and Goblet cells); **(E, F)** immunohistochemistry for Chromogranin A (enteroendocrine cells) and Alcian Blue counterstain (mucus and Goblet cells). Arrow indicates a Chromogranin A positive cell. All representative images were taken at 20x. Scale bars in **(A)** indicate 20 microns and in **(C, E)** indicate 50 microns. Manual quantification of each measure was performed on an average of 30 colonoids per slide, and error bars and IHC/ISH images are representative of the average of biological duplicates from three independent experiments. Statistical significance was determined by one-way ANOVA. **P < 0.01, ***P < 0.005, ****P < 0.001.

**Figure 3:**
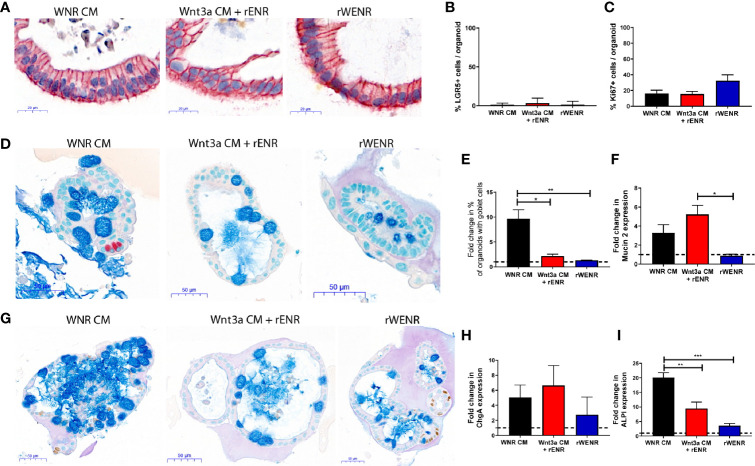
Switching culture conditions from Wnt3a-conditioned medium to Wnt3a free medium induces most efficient differentiation of mouse colonoids. Mouse colonoids derived from C57Bl/6 mice crypts were cultured for two days in WNR CM, Wnt3a CM + rENR or rWENR medium before medium change to rENR or maintenance in original media. After four additional days of culture, colonoids were assayed for markers of epithelial cell types by ISH/IHC and qPCR. **(A, B)** Colonoids were stained with *in situ* hybridization for LGR5 for the quantification of stem cells (brown dots), immunohistochemistry for EpCam (epithelial cells) and hematoxylin (nuclei); **(C)** Quantification of Ki67 histochemistry for measurement of proliferation; **(D)** immunohistochemistry for Chromogranin A (enteroendocrine cells) and Alcian Blue counterstain (mucus and Goblet cells); **(E)** quantification of goblet cells; **(F)** quantification of Mucin 2 mRNA levels; **(G)** immunohistochemistry of Ki67 as a proliferation marker, with an Alcian Blue counterstain (mucus and Goblet cells); **(H)** quantification of chromogranin A immunohistochemistry; **(I)** quantification of enterocytes using mRNA transcript levels of alkaline phosphatase (ALPI). qPCR analysis was performed on biological triplicates for each treatment group at each timepoint. Graphs in **(B, C)** are the average expression by IHC/ISH of the indicated markers in the stem cell conditions from three independent studies. Graphs in **(E, F, H, I)** are the average fold increase in markers by IHC **(E)** or qPCR **(F, H, I)** after differentiation compared to levels in the stem cell conditions from three independent experiments. Scale bars in A indicate 20 microns and in C and E indicate 50 microns. IHC/ISH quantification was performed on an average of 30 colonoids per slide. Graphs and images are representative of the average of biological duplicates from three independent experiments. Statistical significance was determined by one-way ANOVA. *P < 0.05, **P < 0.01, ***P < 0.005.

*CellTiter-Glo^®^ 3D Cell Viability Assay Normalization* (*Routine Passaging*, **Figures 2**, **3**, **5**): To determine a consistent split ratio for the colonoids, colonoids were resuspended in 1 ml of washing media. A 15 ul aliquot of this sample was removed, and then further diluted 1:2 in 15 ul of washing media to make neat 1:2, 1:4, 1:8, and 1:16 dilutions. To a 96-well plate (black walled, clear bottom) 100 ul of thawed *CellTiter-Glo^®^ 3D* (Promega #G9683) + 100 ul of PBS + 10 ul of colonoid suspension or washing media alone was added. The plate was protected from light and incubated at RT for 30 min, with shaking for the first 5 min. During incubation, stock solution of colonoids was kept in washing media on ice. For plating, the solution of colonoids in washing media was spun at 5 min, 600 g, RT couched in a 50 ml conical tube as above. Media was aspirated, and pellet resuspended in the appropriate volume of thawed Matrigel, with the dilution dependent on normalization to a pre-selected value based on luminescence readings. The preferred density for passage and corresponding luminescence value was experimentally evaluated and is dependent on the machine used to read luminescence.

For plating, a drop of Matrigel was added to the middle of each well, and once a plate was complete, it was flipped over and put in 37°C incubator upside down to solidify for 15 min. Solidified Matrigel plugs were then overlaid with 350 ul of growth media/well for 48-well and 500 ul media/well for 24-well. For human colonoids, a consistent Monday-Thursday or Tuesday-Friday split is recommended for colonoid health and to increase reproducibility. For mouse colonoids, splitting Monday, Wednesday, Friday is recommended due to fast growth of the cultures. For routine passaging, colonoids from human and mouse were grown in WNR CM. For other studies, WNt3a CM + rENR, rWENR, rE, rENR medias were used. All media acronyms and components used in this paper are compiled in **Tables 1** and **2**, respectively and the sources of components are given in **Table 3**.

**Table 1:** Colonoid culture media acronyms.

rE	Media supplemented with recombinant EGF
rENR	Media supplemented with recombinant EGF, Noggin, and R-spondin
rWENR	Media supplemented with recombinant Wnt3a, EGF, Noggin, and R-spondin
WNR CM	Wnt3a-Noggin-Rspondin conditioned media
Wnt3a CM + rENR	Wnt3a-conditioned media supplemented with recombinant EGF, Noggin and R-spondin

**Table 2:** Human and mouse colonoid medias.

Colonoid type	Media	Basal Media		Growth factors	Additives
**Mouse**	**rENR**	Advanced DMEM/F12 Hepes, GlutaMAX Pen/Strep N-2, B-27 N-Acetylcysteine		Human R-spondin1 Mouse Noggin Human EGF	
**Mouse**	**rWENR**	Advanced DMEM/F12 Hepes, GlutaMAX Pen/Strep N-2, B-27 N-Acetylcysteine		Human R-spondin1 Mouse Noggin Human EGF Mouse Wnt3a	
**Mouse**	**Wnt3a CM + rENR**	Advanced DMEM/F12 Hepes, GlutaMAX Pen/Strep N-2, B-27 N-Acetylcysteine		Human R-spondin1 Mouse Noggin Human EGF Mouse Wnt3a	
**Mouse** **/Human**	**WNR CM**	Advanced DMEM/F12 20% FBS GlutaMAX Pen/Strep		WNR CM Mouse R-Spondin 3 Mouse Noggin Mouse Wnt3a	TGF-βR Inhibitor ROCK inhibitor** **
**Human**	**rENR + Wnt3a CM**	Advanced DMEM/F12 Hepes, GlutaMAX Pen/Strep N-2, B-27 N-Acetylcysteine	Advanced DMEM/F12 20% FBS GlutaMAX Pen/Strep	Human R-spondin1 Mouse Noggin Human EGF Wnt3a CM	Gastrin Nicotinamide TGF-βR Inhibitor, P38 inhibitor ROCK inhibitor
**Human**	**rWENR**	Advanced DMEM/F12 Hepes, GlutaMAX Pen/Strep N-2, B-27 N-Acetylcysteine		Human R-spondin1 Mouse Noggin Human EGF Mouse Wnt3a	Gastrin Nicotinamide TGF-βR Inhibitor, P38 inhibitor ROCK inhibitor

**Table 3:** Media components and source.

Item	Catalog	Solvent	Final Concentration
Advanced DMEM/F12	ThermoFisher Scientific 12634		
GlutaMAX	ThermoFisher Scientific 35050		1x
Hepes 7.3	ThermoFisher Scientific 15630		1x
Pen/Strep	ThermoFisher Scientific 15140		1x
B-27 Supplement	ThermoFisher Scientific 17504		1x
N-2 Supplement	ThermoFisher Scientific 17502		1x
N-Acetylcysteine	Sigma A9165	Distilled H20	10 uM
Human EGF	ThermoFisher Scientific PMG8043	PBS/0.1% BSA	50 ng/ml
Mouse Noggin	Peprotech 250-38	PBS/0.1% BSA	100 ng/ml
Human R-Spondin-1	R&D 4645-CF	PBS/0.1% BSA	500 ng/ml
Mouse Wnt3a	R&D 1324-CF	PBS/0.1% BSA	100 ng/ml
ROCK Inhibitor (Y-27632)	Sigma Y0503	PBS	10 uM
TGF-βR Inhibitor (SB-431542)	Tocris 1614	DMSO	10 uM
FBS	Sigma F2442		
Matrigel^®^ Growth Factor Reduced (GFR) Basement Membrane Matrix, Phenol Red-Free, *LDEV-Free, 10mL	Corning 356231		
Nicotinamide	Sigma N0636	PBS	10 uM
Gastrin	Sigma G9145	PBS	50 nM
P38 inhibitor (SB-202190)	Tocris 1264	DMSO	10 uM

### Colonoid Passaging for Experiments

For quantification of colonoid viability *via* Cell Titer-GLO 3D and microscopy, colonoids were subcultured as above at a 1:4 split and deposited in 50 ul Matrigel (mouse colonoids) or 15 ul (human colonoids). Colonoids were overlaid with WNR CM, Wnt3a CM + rENR or rWENR. For quantification of colonoid number, after 2 days (mouse colonoids) or 3–4 days (human colonoids) wells were imaged using a Keyence BX-510 microscope with a 2x and 4x objective for colonoid number quantification. Z-stack images with slices every 99uM were obtained from all 4 wells of colonoids for each treatment conditioned at each timepoint. Full focus images were obtained from each Z-stack, and the number or colonoids in each imaged manually counted using ImageJ.

For Cell-titer Glo enumeration, 3 of the 4 wells of colonoids from each growth condition at each timepoint were assayed. Briefly, media was aspirated and the Matrigel plug was incubated in 150 ul cell recovery solution (BD Corning #354253) for 30 min at 4°C. After incubation an equal volume of Cell-titer Glo 3D was added and cells were incubated for a further 30 min at RT, shaking for the first 5 min. 100 ul of the solution was transferred to a 96 well black plate in duplicate and luminescence read on a Biotek Synergy 2. Technical duplicates were averaged to give one value for each well, and for each treatment condition at each timepoint, the average of the three assayed wells is depicted. For passaging, the one remaining well was split 1:4 and processed as above over subsequent passages for five passages.

For qPCR and histology studies, colonoids were grown in WNR CM and passaged as above, deposited in 50 ul Matrigel (mouse colonoids) or 15 ul (human colonoids), and then overlaid with WNR CM, Wnt3a CM + rENR or rWENR media. For mouse colonoids, after 2 days culture media was changed and the colonoids were either put in fresh WNR CM, Wnt3a CM + rENR and rWENR or media was changed to rENR to monitor differentiation. For human colonoids, culture media was change from WNR CM to rE or rENR after 1 day.

**Figure 4:**
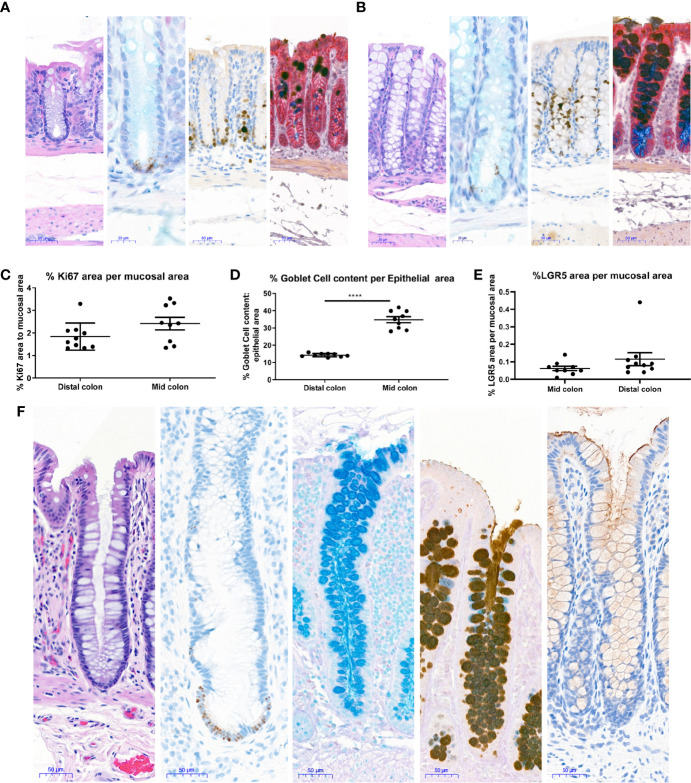
Histological characterization of mouse and human colons. Distal and Mid colons were collected from naïve male C57BL/6N mice and paraffin-embedded for staining. Serial sections were stained for [**(A)** (distal colon) and **B** (mid colon), from left to right] H&E, In situ hybridization for LGR5 as a stem cell marker (black dots at base of crypt) with Alcian blue counterstain for mucus and nuclei, the proliferation marker Ki67 with hematoxylin counterstain (nuclei), dual Immunohistochemistry for Gob5/CLCA1 to label a subset of Goblet cells (dark brown) and GPA33 for epithelial cells (red) was counterstained with Alcian blue-PAS to capture all goblet cells. In **(A, B)**, representative 20x images are shown and scale bars indicate 50 microns for all images except LGR5 ISH, where scale bars indicate 20 microns. Images in **A** are from the distal colon and images in **(B)** are from the mid colon. Stained mouse colons were used for automated quantification to determine the percent Ki67 positive area per mucosal area **(C)**, percent goblet cell area per epithelial area **(D)**, and percent LGR5 positive area per mucosal area **(E)**. Each data point represents the quantification of an image from an individual mouse. Ranges and averages were similar across three independent experiments. Data from one representative study in nine naïve mice is shown. Statistical significance was determined by unpaired t-test. In **(F)**, 20X images show representative staining of a colon from a healthy human for (from left to right) H&E, *in situ* hybridization for LGR5 (black dots) with hematoxylin counterstain (nuclei), Alcian blue to show Goblet cell content, immunohistochemistry for Mucin 2 counterstained with Alcian blue-PAS to capture all goblet cells, and immunohistochemistry for Mucin 1 (brown) with hematoxylin counterstain (nuclei). Scale bars indicate 50 microns for all images. ****P < 0.001.

For viability and apoptosis studies (**Figure 4C**), 6 wells of colonoids per treatment were passaged 1:4 as above, with TGFβ inhibitor, ROCK inhibitor, or both inhibitors in every passaging solution. Colonoids were plated in 10ul of Matrigel in a 96 well plates and overlaid with WNR CM containing the same inhibitors that were present during passaging. To measure apoptosis, after 24 h media was removed and replaced with 100 ul of Caspase-Glo^®^ 3/7 Assay reagent (Promega G8090), followed by shaking incubation for 30 mins before reading luminescence on a Biotek Synergy 2. To measure viability, after 72 h media was removed and replaced with 100 ul of CellTiter-Glo^®^ 3D reagent (Promega #G9683), followed by shaking incubation for 30 mins before reading luminescence on a Biotek Synergy 2.

### qPCR Analysis

**In each study at each timepoint,** RNA was extracted from 3 wells of colonoids embedded in Matrigel using Qiagen RNAeasy mini kit (Qiagen #74104) according to the manufacturer’s instructions. RNA was quantified using a Nanodrop One (ThermoFisher Scientific), and 2 ug of total RNA was reverse transcribed into cDNA using the High-Capacity cDNA Reverse Transcription Kit (ThermoFisher Scientific #4368813). RT-PCR was performed on ViiA7 real-time PCR system using TaqMan Assays-on-Demand™ Gene Expression assays. The comparative CT (DDCT) method was used to determine the relative expression of target gene compared to 18 s as a housekeeping gene.

### Necroptosis Assays

Human colonoids were grown in WNR CM and passaged as above. After 3 days in culture, media was changed to WNR CM containing DMSO (vehicle) or 10 ng/ml TNFα (R & D Systems, Minneapolis, MN). + 80 uM pan caspase inhibitor z-VAD-FMK (zVAD, R & D Systems) + 2 uM AZD 5582 second mitochondria-derived activator of caspase (Smac) mimetics (Tocris, Minneapolis, MN). The main endpoints of interest were measured 24 h later. Cell viability was determined with imaging using a BZ-X microscope (Keyence, Itasca, IL) and CellTiter-Glo 3D reagent (Promega, Madison, WI). Supernatants were collected for HMGB1 analysis by ELISA (IBL International/Tecan, Hamburg, Germany) and cytokine analysis by V-plex immunoassay (Meso Scale Diagnostics, Rockville, MD). For protein expression, cells were lysed with lysis buffer (Cell Signaling Technologies, Danvers, MA) plus HALT protease and phosphatase inhibitor cocktail (ThermoFisher Scientific). Protein concentration was determined with BCA kit (ThermoFisher Scientific). Phosphorylated MLKL (pMLKL) was determined by western blotting using an anti-pMLKL (Ser358) antibody (Cell Signaling Technology, 91689, clone D6H3V). Anti-b-actin antibody (Cell Signaling Technologies, clone 8H10D10) was used as a loading control.

### Histopathology

At necropsy, the distal 5 cm of colon was opened, fecal pellets removed with forceps, and the mucosa gently flushed with saline. Colons were cut into 2x 2.5 cm lengths, laid flat between two biopsy sponges in a cassette and immersion fixed in 10% neutral buffered formalin. After 48 h fixation, colon samples were routinely processed, trimmed, embedded on edge into paraffin blocks and sectioned at 5 um onto charged glass slides.

### Immunohistochemistry and *In Situ* Hybridization

Tips and tubes were precoated with 10% BSA to avoid sticking and improve colonoid recovery. Matrigel plugs were disrupted by scraping with a P1000 pipette and transferred to 15 ml conical tubes, spun down at 600 g, 5 min, RT and washed 1x with dPBS. After a second spin as above, supernatant was removed and colonoids were resuspended in 2 ml of 10% NBF (Harleco # 65346-88) at RT for 30 min. After washing in PBS, colonoids were incubated twice for 2 min each at RT in 50% ethanol, followed by a 30 min RT incubation in Eosin to label the colonoids and ease subsequent identification during embedding. Following two washes in PBS, colonoids were resuspended in 100 ul of liquid Histogel (ThermoFisher Scientific #HG-4000-012) in the base of a 1.5 ml tube. Resuspend colonoids were then placed on ice to hasten Histogel solidification, before the Histogel was transferred to a cassette and submerged in NBF. After overnight fixation, samples were embedded in paraffin and sectioned for ISH and IHC staining.

All immunohistochemistry (IHC), *in situ* hybridization (ISH), and multiplexing was performed on the Leica Bond RX. For immunohistochemistry, tissue sections were subjected to either citrate or EDTA heat-induced epitope retrieval, incubated with the primary antibody, detected with either Leica’s HRP or AP polymer, and visualized with either DAB (Leica’s Bond Polymer Refine Detection kit) or Red (Leica’s Bond Polymer Refine Red Detection kit). All detection reagents and working concentrations are summarized in **Table 4**.

**Table 4:** Antibodies for IHC and probes for ISH.

Stain	Species	Vendor/Catalogue number	Working concentration
MUC1 (Rb anti-hu)	human	Millipore Sigma Cat #HPA008855 Lot #96984	0.3 ug/ml
Rabbit IgG	human	Vector Laboratories Cat #I-1000 Lot #X0720	0.3 ug/ml
MUC2 (Rb mAb anti-hu)	human	Abcam Cat #ab134119 Lot #GR155812-1	0.0075 ug/ml
Rabbit (DA1E) mAb IgG	human	Cell Signaling Cat #3900 Lot #30	0.0075 ug/ml
Ki67	human and mouse	Roche Cat #790-4286 Lot #Y13569, D11266	2.0 ug/ml (both human and mouse)
LGR5 ISH	human and mouse	ACD Cat #311028 Lot #17354B, 17094A, 17065A (human) Cat #312178 Lot #15266A (mouse)	
Chromogranin A	mouse	Novus Biologicals Cat #NB120-15160 Lot #131030LVD	2.5 ug/ml
GPA33	human and mouse	Origene Cat #TA349330 Lot #D9290AB0001, D913AB0001 (human) Lot #D502AA0011 (mouse)	3.8 ug/ml (human) 10 ug/ml (mouse)
Epcam	mouse	Novus Biologicals Cat #NBP2-27107 Lot #06204307A-2	7.0 ug/ml
Gob5	mouse	Abcam Cat #ab46512 Lot #GR3188521-1	0.5 ug/ml
IBA1	human	Wako Cat# 019-19741	0.05 ug/ml

For multiplexing, sequential staining was used. Tissue sections were subjected to either citrate or EDTA heat-induced epitope retrieval, incubated with the first primary antibody, detected with Leica’s HRP polymer and DAB (Leica’s Bond Polymer Refine Detection kit). Immediately following the DAB step, the second primary antibody was applied, detected with Leica’s AP polymer, and visualized with Red (Leica’s Bond Polymer Refine Detection kit).

For *in situ* hybridization, ACD’s RNAscope 2.5 LS Reagent kit was used. The 2.5 LS Assay was used which incorporates a series of proprietary reagents and visualization with ACD’s DAB; epitope retrieval was done with both Leica’s EDTA and ACD’s Protease. For the multiplexed ISH/IHC, ACD’s RNAscope 2.5 LS Reagent kit was used as described above, however, it was combined with sequential staining. After the DAB, the primary antibody was applied, then detection with Leica’s AP polymer and visualization with Red (Leica’s Bond Polymer Refine Red Detection kit).

For manual analysis (mouse colonoid samples) of Ki67, LGR5 and Alcian blue images were scanned on the Vectra Polaris at 20x and unmixed. For counting, images were opened in ImageJ and counted using the cell counter plugin. For Ki67 the number of Ki67 positive and Ki67 negative nuclei were enumerated for each colonoid. Only visible, stained nuclei were counted, and any brown staining in the nuclei was considered positive. For LGR5 ISH, one spot per cell was considered an LGR5-positive cell and results are presented as percent LGR5-positive cells/individual colonoids. For Goblet cell quantification, colonoids were scored as Goblet cell positive, defined as Alcian blue staining in the cell layer. For manual counting, an average of 30 colonoids were analyzed per slide (range 20–50).

For automated analysis of LGR5, Ki67, Mucin 2, Glycoprotein A33 (GPA33), and Alcian blue on human colonoids histology slides were scanned on Vectra Polaris at 20x. Next, 15 40x High power fields per slide were randomly selected and imaged into multispectral images. Spectral library was applied, and images were unmixed in Inform. InForm was trained based on the spectral library to segment and identify the cell layer. Separate algorithms were developed for Ki67, Mucin 2, GPA33, LGR5 and Alcian blue cell/object analysis. Each 40x image contained an average of 25 colonoids, and for each slide the output from each 40x image was averaged to give one value of % positive cells per colonoid or per images for every slide. For Mucin 1 analysis, slides were digitized on a Pannoramic 250 Flash III scanner (3D HISTECH) and transferred to Visiopharm for image analysis. The Mucin 1 staining was defined as the area of Mucin1 immunopositive pixels per colonoid area pixels after luminal white space was subtracted. Mucin 1 analysis was applied to the entire slide image, each of which contained an average of 570 colonoids.

For automated analysis of LGR5, Ki67, Gob5, GPA33, and Alcian blue on mouse colons, slides were digitized on a Panoramic 250 Flash III scanner (3D HISTECH) and transferred to Visiopharm for image analysis. The Ki67 and LGR5 staining was defined as the area of immunopositive pixels per mucosal area. For goblet cell quantification, the epithelial area was defined as the area of the mucosa which was GPA33 positive, and within this area, the area of Gob5 positive and Alcian blue positive area was combined in order to define the total % goblet cell positive area per epithelial area. The distal colon was defined as the ~2.5 cm of colon immediately adjacent to the anus and containing longitudinal mucosal folds. The mid colon was defined as the region from 2.5 to 5 cm orad to the anus, and containing no mucosal folds. Analysis was applied to a defined 15 mmx1.5 mm ROI fin the mid or distal segment of colon for each animal.

### Statistics

Experiments were analyzed using Prism (v. 7.0d, GraphPad). For all figures, data were analyzed by one-way analysis of variance with Tukey’s post-tests. In all the analyses, P <0.05 was considered significant. In the figures, single asterisk (*) indicates P <0.05, double asterisks (**) indicates P <0.01, and triple asterisks (***) indicates P <0.005, and quadruple asterisks (****) indicates P <0.001.

## Results

### Wnt3a-Conditioned Medium Is Essential for the Long-Term Survival of Mouse Colonoid Cultures

Mouse colonoids (going forward, we will use this term because our cultures are derived from colonic adult stem cell) were grown for five passages comparing four different growth media conditions: Wnt3a-Noggin-Rspondin conditioned media (WNR CM, **Figure 1A** first row of images), Wnt3a-conditioned media supplemented with recombinant EGF, Noggin and R-spondin (Wnt3a CM + rENR, **Figure 1A** second row), media with recombinant Wnt3a, EGF, Noggin, and R-spondin (rWENR, **Figure 1A** third row), or the latter medium without Wnt3a (rENR, **Figure 1A** fourth row). All media acronyms and components used in this paper are compiled in **Tables 1** and **2**, respectively. At every passage, 2 wells of colonoids were quantified using the Cell Titer-Glo 3D luminescent cell viability assay and the remaining wells were imaged before further passaging. After the first passage, a reduction in the number and size of colonoids was apparent in cultures with rWENR or rENR medium (**Figure 1A**). By the fifth passages, no surviving colonoids were observed in either of the latter two culture conditions. Colonoid-forming efficiency was determined by measuring cell viability with Cell Titer-Glo (**Figure 1B**) and counting colonoid numbers per power field by light microscopy (**Figure 1C**). This confirmed that colonoid growth in rWENR and rENR medium is substantially reduced compared to culture in the other two media in which colonoid numbers and viability remained steady and comparable over the entire passage time (**Figures 1A–C**). No significant differences in viability (**Figure 1B**) or number of colonoids (**Figure 1C**) was detected between WNR CM and Wnt3a CM + rENR medium indicating that both media sustained long-term culture of mouse colonoids in a comparable manner.

This analysis not only confirmed the critical role of Wnt-signaling for the survival of colonoid cultures but also demonstrated that recombinant Wnt3a by itself cannot substitute for Wnt3a-conditioned media with regards to providing optimal growth conditions in mouse colonoid cultures. Since Cell Titer-Glo quantification and colonoid numbers correlated highly in all conditions (2x or 4x images, **Supplementary Figure 1**), subsequent studies relied exclusively on Cell Titer-Glo as a measure of colonoid forming efficiency, viability, and growth.

### Recombinant Wnt3a Fails to Support Stem Cell Survival and Proliferation in Mouse Colonoids

Wnt3a has been described as an essential niche component for maintaining the proliferation of LGR5-positive stem cells in intestinal colonoids. It is furthermore well appreciated that long-term cultures depend on the maintenance of proliferative stem cells. Accordingly, the presence of proliferative markers directly correlates with the absence of cellular markers for terminally differentiated epithelial cells in propagating cultures. We next attempted to delineate the biological mechanisms underlying the observation that conditioned media derived Wnt3a, but not recombinant Wnt3a, promoted the long-term survival of mouse colonoids.

When grown in any Wnt3a containing media, colonoids have a spheroid appearance by brightfield microscopy and are histologically characterized by a thin epithelial layer and a hollow, spherical lumen (**Supplementary Figure 2A**, first two panels). Upon Wnt3a withdrawal, the colonoid epithelial layer increases in thickness, and differentiated cell types such as Goblet cells appear (**Supplementary Figure 2**, second two panels). Mouse colonoids were grown for nine passages in WNR CM, before dissociation into single cells and culture for six days in WNR CM, Wnt3a CM + rENR, or rWENR (**Figures 2A, C**, and **E**). Phenotypic differences were immediately apparent between the culture conditions. Colonoids grown in rWENR had lost the spheroid phenotype associated with growth in stem cell enriching conditions. We next quantified the stem cell compartment using expression of the intestinal stem cell marker Leucine-rich repeat-containing G-protein coupled receptor 5 (LGR5) and assessed proliferation with Ki67 expression. Highest expression of LGR5 was detected with WNR CM medium [*in situ* hybridization (ISH) in **Figure 2A**, quantification of ISH in **Figure 2B**, and qPCR in **Figure S2B**]. The Wnt3a CM + rENR condition presented with a substantial decrease in the LGR5-positive compartment compared to WNR CM but the most dramatic loss of the LGR5 signal was observed with the rWENR medium (**Figures 2A, B**, and **Supplementary Figure 2B**) in line with the phenotypic alteration of loss of cystic colonoids in the rWENR condition. A significant reduction in the Ki67 signal was observed in rWENR and Wnt3a CM + rENR media compared to WNR CM (**Figures 2C, D**). Surprisingly, the proliferation as assessed by Ki67 did not show any statistically significant differences between in rWENR and Wnt3a CM + rENR media (**Figure 2D**). To further understand if global alteration in the Wnt signaling pathway accounts for the differences between media conditions, we tested the expression levels of Axin2, an established Wnt target gene. No significant differences in Axin2 mRNA levels were detected between WNR CM and Wnt3a CM + rENR (**Supplementary Figure 2C**). In contrast, cultures supported by recombinant Wnt3a (rWENR medium) showed significantly lower levels of Axin2 (**Supplementary Figure 2C**). A trend towards lower levels of Axin2 was also observed in rWENR compared to Wnt3a CM + rENR (**Supplementary Figure 2C**). These results indicate that rWENR is less capable of maintaining Wnt3a dependent stemness signals compared to WNR CM or Wnt3aCM + rENR.

To next assess spontaneous differentiation in proliferation supporting culture conditions, presence of Goblet cells in colonoids was quantified using Alcian blue staining for acidic Mucins (**Figure 2E** and quantification in **Figure 2F**). Inversely to LGR5 expression, the lowest number of colonoids staining positive for Goblet cells was observed with WNR CM. Significant increases in Goblet cells were found in the two other conditions with the highest numbers detected in rWENR medium. Quantification of Mucin 2 (Muc2) RNA levels (**Supplementary Figure 2D**) supported the observation that the highest spontaneous differentiation occurred in rWENR media. We also analyzed expression levels of Chromogranin A (ChgA) as a marker of enteroendocrine cells, another differentiated epithelial cell type found in colonoid cultures (**Figure 2E**, indicated by arrow, **Supplementary Figure 2E**). In line with our earlier observations, the highest number of ChgA transcripts was detected in rWENR medium indicating that this medium induces the highest degree of differentiation for multiple epithelial cell types.

These data show that recombinant Wnt3a is unable to support the persistence of a stem cell niche in mouse colonoids compared to Wnt3a-conditioned media. Both the loss of stem cells and the increase in spontaneously differentiating cells observed after growth in rWENR likely contribute to the inability of this medium to sustain long-term growth in mouse colonoid cultures. In addition, the decrease in proliferative stem cells observed with Wnt3a CM + rENR suggests that, while this medium appears sufficient to support long-term survival of mouse colonoids, it lacks the essential key factor(s) for stem cell self-renewal that are present in WNR CM.

### Switch of Culture Conditions From Wnt3a-Containing to Wnt3a-Free Medium Induces Differentiation of Mouse Colonoids

We so far showed that colonoids grown in Wnt3a-containing media (WNR CM) are stem cell-enriched and, next, wanted to study the effect of Wnt removal on the cultures. According to the literature, an increase in differentiated epithelial cell types including Goblet cells, enteroendocrine cells, and enterocytes was expected. Since the colonoids grown with Wnt3a-conditioned media supplemented with recombinant EGF, Noggin and R-spondin (Wnt3a CM + rENR) and media with recombinant Wnt3a, EGF, Noggin, and R-spondin (rWENR) were already enriched in differentiated cells compared to the Wnt3a-Noggin-Rspondin conditioned media (WNR CM), it was important to also define the effect of Wnt-removal on further differentiation from those cultures. For all growth conditions, Wnt-removal was achieved by the switch of culture conditions to Wnt3a-free medium.

After two days of growth in Wnt3a-containing media, the three different mouse colonoid cultures were switched to culture in rENR media, which lacks any source of Wnt3a (referred to as differentiation medium in the following sections) for another four days. Across all growth conditions, removal of Wnt resulted in a significant loss of the LGR5 signal (ISH in **Figure 3A**, quantification in **Figure 3B**) and the Ki67 signal (quantification of immunohistochemistry in **Figure 3C**). When comparing the cellular composition of colonoids grown in differentiation medium (rENR) to the original stem-cell-maintaining conditions (WNR CM, Wnt3a CM + rENR, or rWENR), colonoids differentiated after growth in WNR CM display a significant increase in Goblet cells, as indicated by an increase in number of Alcian blue positive cells (**Figures 3D, E, G**) and Muc2 RNA levels (**Figure 3F**). Similar increases in enteroendocrine (ChgA, **Figure 3H**) and enterocyte markers (ALPI, **Figure 3I**) were also observed after differentiation from WNR CM.

We already showed that culture in WNR CM provides an environment more enriched in LGR5-positive stem cells and proliferation. This set of data demonstrated, that upon Wnt removal, colonoids grown in WNR CM show a higher percentage of differentiated cells that colonoids differentiated from Wnt3a CM + rENR or rWENR, indicating that WNR CM is more conducive to promoting differentiation. These observations support the overall conclusion that colonoids expanded in and differentiated from WNR CM medium grow more efficiently and most closely reflect the composition of the intestinal epithelium *in vivo*.

### Histological Characterization of Mouse and Human Colons

To understand if the epithelial compartment of *in vitro* generated mouse colonoids was representative of the *in vivo* situation in healthy tissue, staining and quantification of different epithelial cell types were performed in the colons of naïve mice. Stains were chosen to enable quantification of both the proliferating cells in the base of the crypt as well as mature cells types such as Goblet cells. H&E staining showed no gross differences between mid and distal colon (**Figures 4A, B**). In line with the published literature LGR5+ stem cells were observed exclusively in the crypt base in both the mid and distal colon (**Figures 4A, B**). Proliferating Ki67 immunopositive cells were only present in the crypt base (**Figures 4A, B**), as expected for this highly proliferative compartment. Upon quantification, no significant differences were detected in the percentage of LGR5 positive mucosa or the percentage of Ki67 positive mucosa between the distal and mid colon (**Figures 4C, E**). Detailed quantification showed that Goblet cells and mucus make up between 13–18% (Average 14.2%, Std 0.98) of the epithelial area in the distal colon while this number increased to 28–42% (Average 34.8% Std 5.18) in the mid colon (**Figure 4D**). Since the higher abundance of Goblet cells *in vitro* compares to the mid colon *in vivo*, we conclude that we are modeling the epithelium of the mid rather than the distal colon in our cultures. Finally, in a parallel approach using tissue from a healthy human donor (**Figure 4F**), we confirmed that the Goblet cell numbers in the human transverse colon appear to be comparable to the murine mid colon. Therefore, we next sought to understand whether the culture conditions optimized for mouse colonoids could be applied to the generation of human colonoids.

### Long Term Survival of Human Colonoids Depends on Optimized Wnt Signaling Conditions

**Figure 5:**
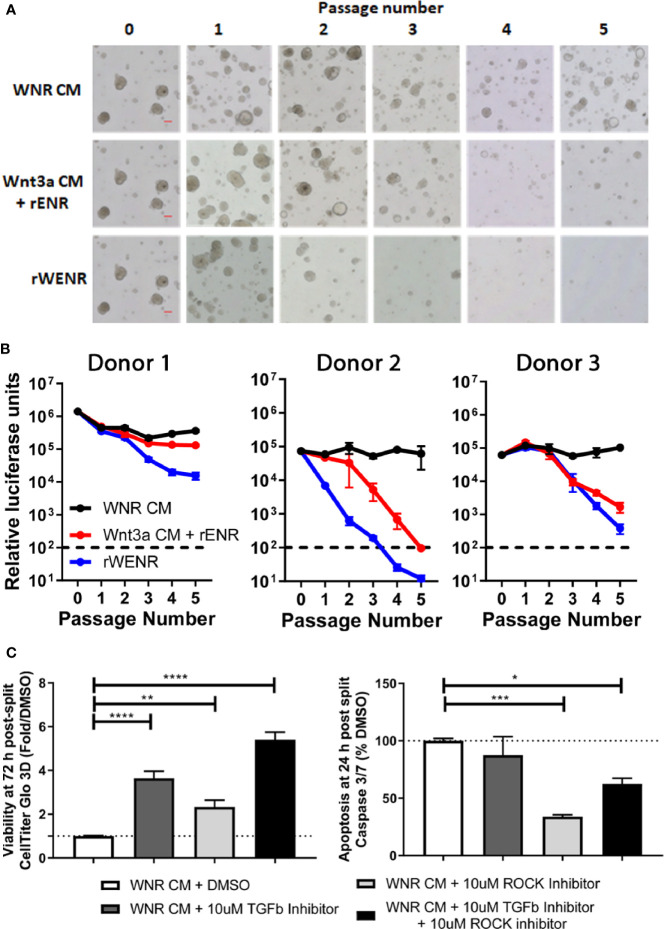
Wnt conditions optimized in mouse colonoids allow for the long-term survival of human colonoids. Survival of human colonoids derived from three healthy donors was compared in different culture conditions over five passages. Media culture conditions from the top: Wnt3a-Noggin-Rspondin conditioned media (WNR CM), Wnt3a-conditioned media supplemented with recombinant EGF, Noggin and R-spondin (Wnt3a CM + rENR), and media with recombinant Wnt3a, EGF, Noggin, and R-spondin (rWENR). **(A)** Representative bright field images for each growth condition medium at every passage from colonoids derived from Donor 1. Scale bars indicate 100 microns. **(B)** Viability was assessed before each passage using the Cell Titer-Glo 3D luminescent cell viability assay in colonoid cultures from all three donors (WNR CM as black lines, Wnt3a CM + rENR as red lines, and rWENR medium as blue line). For each donor and treatment at each timepoint, 3 wells of colonoids were quantified by Cell Titer-Glo 3D and error bars represent the average. Representative images and survival from two independent experiments is shown. **(C)** Impact of TGFb inhibitor and ROCK inhibitor on organoid viability and apoptosis during splitting were assessed by Cell Titer-Glo 3D and Caspase-Glo 3/7 Assay, respectively. Data from Donor 2 is shown and is representative of studies done is Donor 1 and Donor 2. Error bars are representative of the average of biological triplicates from two independent experiments, one in Donor 1 and one in Donor 2. Statistical significance was determined by one-way ANOVA. *P < 0.05, **P < 0.01, ***P < 0.005, ****P < 0.001.

Following the experimental strategy delineated for studying murine colonoids, human colonoids were grown for five passages in WNR CM, Wnt3a CM + rENR or rWENR. To address donor variability in humans, colonoid lines were derived from non-inflamed biopsies collected during routine colonoscopies from three individuals (14). Representative images from Donor 1 (**Figure 5A**) show that a reduction in growth was detected as early as passage two with rWENR medium while growth in Wnt3a CM + rENR appeared delayed at passage four. Quantification of cell viability as a measure for organoid forming efficiency across all three donors showed a reduction with the Wnt3a CM + rENR (**Figure 5B**, red lines) and the rWENR medium (**Figure 5B**, blue lines) compared to WNR CM (**Figure 5B**, black lines). Donor to donor variability in the ability of Wnt3a CM + rENR and rWENR to support colonoid growth was observed with Donor 1 overall responding with better growth than Donor 2 and Donor 3 (**Figure 5B**). However, WNR CM medium supported colonoid growth to the same level across all three donors.

When described as a media to support colonoid growth, WNR CM has two common additives, TGFβ inhibitor and ROCK inhibitor (14, 16). Addition of ROCK inhibitor has been described to increase the survival of dissociated embryonic stem cells, and therefore it is anticipated that ROCK inhibitor will reduce apoptosis during colonoid passaging (2, 10, 21). TGFβ inhibitor has also been described to be necessary for the long-term culture of colonoids (14). In order to understand the impact of the inhibitors on apoptosis and viability, stem cells were passaged in the presence of both inhibitors, no inhibitors or either inhibitor alone, followed by plating and growth in WNR CM with both inhibitors, no inhibitors, or either inhibitor alone. Consistent with the literature, we found that the addition of ROCK inhibitor, but not TGFβ inhibitor led to a significant decrease in apoptosis in the cultures at 24 h post plating (**Figure 5C**). However, significance increases in viability as measured by Cell-titer GLO 3D were observed after treatment with either inhibitor alone or both inhibitors 72 h after plating (**Figure 5C**). Although the addition of ROCK inhibitor alone led to a larger decrease in apoptosis than the combined inhibitor treatment, the fact that viability was highest after combined treatment supports the addition of both inhibitors to WNR CM for optimal growth. Since WNR CM plus inhibitors could decrease apoptosis, increase viability, and counteract human donor variability, this medium was selected for colonoid growth of human cultures going forward.

### Optimal Differentiation of Human Colonoids Requires a Change in Culture Condition to Wnt3a-, Noggin-, and R-spondin-free Medium

**Figure 6:**
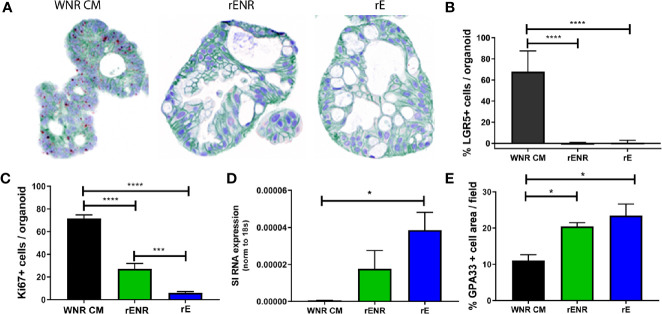
Switch from a proliferative to a differentiation program in human colonoids is achieved by the combined removal of Wnt3a, Noggin, and R-spondin in cultures. Human colonoids derived from the Donor 1 biopsy were cultured for one day in WNR CM, and either kept in this medium or switched to rENR and rE as differentiation culture condition. After 3 additional days, colonoids were assayed by histology and qPCR for proliferation and differentiation markers. **(A)** In situ hybridization for LGR5 (stem cells), immunohistochemistry for GPA33 (all epithelial cells) and Hematoxylin (nuclei) and **(B)** quantification of LGR5 positive stem cells; **(C)** Quantification of Ki67 immunohistochemistry (representative images of stain shown in **A (D)** Sucrose isomaltase mRNA transcript levels; **(E)** quantification of GPA33 positive cells area from images in **(A)**. Error bars for qPCR are representative of biological triplicates from two independent studies. For automated analysis of LGR5, Ki67, and GPA33, data is the average number for three slides and is representative of two independent studies. For each slide, the average number was determined by collecting and analyzing 15 random 40x images (containing an average of 25 colonoids each) to determine % positive cells per colonoid or per image. Statistical significance was determined by one-way ANOVA.

To define the optimal conditions for the differentiation of human colonoids, human colonoids from Donor 1 were cultured for one day in WNR CM medium. Next, culture conditions were switched to rENR or rE medium compared to maintenance of cultures in WNR CM. After three additional days of culture, colonoids were assayed by histology and qPCR for markers of epithelial cell types. LGR5-positive cells were highly abundant in colonoids grown in WNR CM but completely absent in rENR or rE medium (ISH in **Figure 6A**, quantification in **Figure 6B**). Proliferation, as assessed by Ki67 IHC, was highest with WNR CM, some signal was found in rENR but no proliferation was detected in rE medium (**Figure 6C**). Similarly, when comparing rENR and rE, with regards to the ability to induce enterocyte as monitored by sucrose isomaltase (SI) and Glycoprotein A33 (GPA33) production, a much higher signal was detected with rE (**Figures 6D, E**).

**Figure 7:**
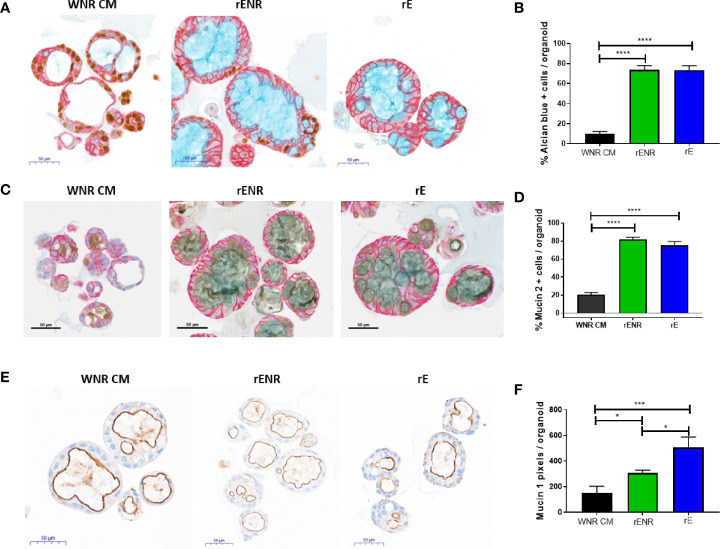
Phenotypic characterization of mucus and goblet cells in human colonoids after removal of Wnt3a, Noggin, and R-spondin from cultures. Human colonoids derived from the Donor 1 biopsy were cultured for one day in WNR CM, and either kept in this medium or switched to rENR and rE as differentiation culture condition. After three additional days, phenotypic characterization of mucus and goblet cells was performed by histology and qPCR. **(A)** Immunohistochemistry for Ki67 (proliferation), GPA33 (all epithelial cells) and Alcian blue staining for goblet cells and mucus and **(B)** quantification of goblet cells; **(C)** Immunohistochemistry for Mucin 2, GPA33 (all epithelial cells) and Hematoxylin (nuclei); and **(D)** quantification of Mucin 2 positive Goblet cells; **(E)** Immunohistochemistry for Mucin 1 with Hematoxylin (nuclei) and **(F)** quantification of Mucin 1 positive cell area. For automated analysis of Alcian blue and Mucin 2 data is the average number for three slides and is representative of two independent studies. For each slide, the average number was determined by collecting and analyzing 15 random 40x images (containing an average of 25 colonoids each) to determine % positive cells per colonoid or per image. The Mucin 1 staining was defined as the area of Mucin 1 immunopositive pixels per colonoid area pixels after luminal white space was subtracted. Mucin 1 analysis was applied to the entire slide image, each of which contained an average of 570 colonoids. Scale bars indicate 50 microns. Statistical significance was determined by one-way ANOVA.

Differentiation of goblet cells and mucus production were then investigated for further phenotypic characterization of the human colonoids in rENR and rE differentiation media. Goblet cell numbers were again quantified as Alcian blue positive cells per colonoid (**Figure 7A**, quantification in **Figure 7B**) and by the number of Muc2 positive cells per colonoid (**Figure 7C**, quantification in **Figure 7D**). Both Goblet cell detection methods showed a significant increase in rE and rENR media. In addition to secreted mucus produced by Goblet cells, all intestinal epithelial cells produce membrane bound Mucins which are an integral part of maintenance of the intestinal barrier and have signaling functions (22). Additionally, quantification of Mucin 1 (Muc1), a representative membrane bound Mucin, in differentiated colonoids showed the highest expression in rE compared to WNR CM and rENR (**Figure 7E**, quantification in **Figure 7F**). Since the intestinal epithelium balances membrane-bound and secreted Mucins for protection purposes, growth conditions, like rE, which show high levels of both of Mucin products are considered superior to conditions in which only one type of Mucin is increased. In conclusion, these data show that optimal differentiation of human colonoids is obtained with rE differentiation medium, which lacks Wnt3a-, Noggin-, and R-spondin.

### Organoids as a Model for Intestinal Necroptosis

**Figure 8:**
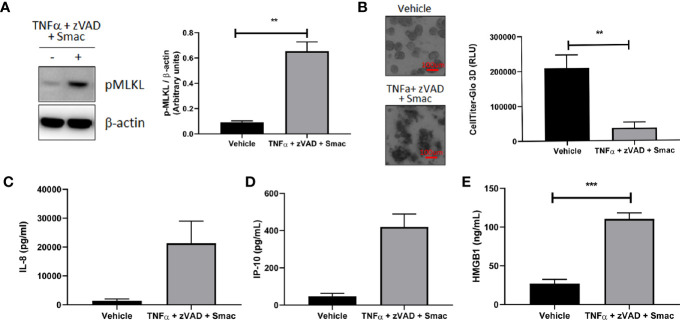
Organoids as a model for intestinal necroptosis. Human colon organoids derived from Donor 1 and Donor 3 were treated with either vehicle or TNFα 10 ng/ml + zVAD 80 uM + Smac 2 uM for 24 h. **(A)** pMLKL protein expression was assessed by western blot analysis. Representative blots probed for pMLKL and β-actin obtained with Donor 1 are shown. Densitometric analysis was performed from five independent experiments, four in Donor 1 and one in Donor 3. Data is expressed as mean ± SEM and shows unpaired t-test; **(B)** Cell viability was assessed by 4X bright field imaging and CellTiter-Glo 3D luminescent assay. Representative images obtained with Donor 1 are shown. Scale bars indicate 100 microns. Data in graph is expressed as mean ± SEM and shows unpaired t-test from six independent experiments, four in Donor 1 and two in Donor 3; **(C–E)**, HMGB1 **(C)**, IP-10 **(D)**, and IL-8 **(E)** production was measured in culture supernatants using ELISA **(C)** and MSD **(D, E)**. HMGB1 data generated with Donor 1 is expressed as mean ± SEM and shows unpaired t-test from four independent experiments. IP-10 data generated with Donor 3 is expressed as mean ± SEM and shows unpaired t-test from two independent experiments. IL-8 data is expressed as mean ± SEM and shows unpaired t-test from three independent experiments, one in Donor 1 and two in Donor 3.

We next explored the utility of our human organoid systems as physiologically relevant models that recapitulate key features of the pathophysiology of intestinal inflammatory diseases that involve immune cell and epithelial barrier dysfunction. Because dysregulated necroptosis can affect both immune cells and epithelial cells in the pathogenesis of intestinal inflammatory diseases, we chose to investigate whether this process can occur in our colonoid model system. To this end, we first evaluated the ability of human colonoids to undergo necroptosis by assessing the effects of classic necroptosis inducers TNFα + Smac + zVAD on phosphorylation of MLKL, which is a critical step for necroptosis execution (23). Western-blot analysis demonstrated that the protein levels of phosphorylated MLKL were increased in organoids stimulated with TNFα + Smac + zVAD (**Figure 8A**), whereas organoids stimulated with TNFα alone did not demonstrate increased MLKL phosphorylation (data not shown). This data indicated that the necroptosis pathway can be specifically initiated in organoids. In agreement with this observation, we showed that TNFα + Smac + zVAD led to cell death as assessed by the presence of colonoids with disrupted morphology in the brightfield microscope, and these effects were quantified using the CellTiter-Glo assay (**Figure 8B**). Because necroptosis has been proposed to play a role in the development of intestinal inflammation (23), we next tested whether TNFα + Smac + zVAD could impact the levels of inflammatory mediators produced by colonoids. Interestingly, treatment with these necroptosis inducers led to increased secretion of the damage-associated molecular pattern (DAMP) molecule HMGB1 (**Figure 8C**), and the chemokines IP-10 (**Figure 8D**) and IL-8 (**Figure 8E**). Overall, this dataset suggests that colonoids represent a novel *in vitro* model of necroptosis that captures the characteristics features of cell death and inflammation. As such, these colonoids provide a unique opportunity to explore the role of necroptosis in the pathophysiology of intestinal inflammatory diseases such as IBD.

## Discussion

The development of *in vitro* organoid cultures offers researchers remarkable access to new model systems across many areas of biomedical research. More specifically for this study, the increasing number of different enteroid, colonoid, and organoid models has helped to rapidly advance our understanding of the role of the polarized epithelium in human intestinal physiology and pathophysiology. However, the field currently lacks detailed systematic approaches that compare culture conditions and their effects on proliferation and differentiation in adult stem cell derived colonoids side by side. To address this knowledge gap, we here focused on comparing the long-term growth and functional differentiation of human and mouse colonoids with the purpose of establishing colonoid cultures with efficient growth throughout passaging and narrowly defined reproducible physiological characteristics as a broadly applicable experimental platform.

In this study, we limited our comparisons to the use of stem cell-derived cultures and analyzed the effect of three commonly used general media compositions as follows: 1) media made with all recombinant growth factors as described by Sato and Clevers (referred to as rWENR) (10, 11); 2) media where all growth factors are recombinant except for Wnt3a, which was derived from conditioned media from an L-cell line from ATCC (Wnt3a CM + rENR), and 3) media that relies on conditioned media as described by VanDussen and Stappenbeck (WNR CM) (14). Following established culture strategies that narrowly monitor the density of colonoids in culture, we confirmed the superiority of Wnt3a-conditioned media to media supplemented with recombinant Wnt3a for maintenance of the proliferative program in murine colonoids. Amongst all culture conditions tested, WNR CM was also the best in maintaining growth throughout passaging in human colonoid cultures and, importantly, was able to counterbalance donor-to-donor variability in organoid-forming efficiency. It is also important to note that commercially available “ready-to-use media” do exist and may represent a convenient option for the expansion of intestinal organoids that can save time and resources at the beginning of a project. However, their proprietary nature limits the practical applicability for studies of the molecular players involved in epithelial processes and functions and may present Intellectual Property challenges in drug discovery.

The optimal long-term culture conditions were identical for human and murine colonoids, but species-specific effects were observed when comparing differentiation conditions. In murine colonoids, switching cultures to Wnt3a-free conditions induced fully differentiated colonoids based on cellular markers of functional enterocytes, enteroendocrine cells, and Goblet cells. As expected, this switch to a differentiation program correlated inversely with a decrease in the proliferating stem cell compartment. In contrast, a similar level of differentiation was only achieved with culture conditions devoid of Wnt3a, Noggin, and R-spondin for human colonoids. This observation stresses the importance for performing detailed comparative studies on culture conditions because species-specific cellular differentiation programs of epithelial stem cells might result in cultures of varying epithelial type composition. Such differences might consequently hamper interpretation of results and their translation across model systems established from tissue sources from mice, rats, primates, or humans if not addressed by tailored culture conditions.

The Goblet cell compartment appeared also clearly affected when comparing media effects on epithelial differentiation between mice and humans (24). Goblet cells are glandular epithelial cells with the main function to produce and secrete the major components of the mucus layer. At the epithelial barrier of the large intestine, which colonoid cultures represent, the inner mucus layer separates the commensal bacteria from the epithelium and the outer colonic mucus layer is the natural habitat for the commensal bacteria (25). Both mucus layers appear functionally impaired in diseases that are associated with colonic mucosal inflammation (26). Since Goblet cells are a main mucus producing cell type, they are considered critical regulators of intestinal immune homeostasis, host-pathogen/commensal interactions, and gatekeepers of innate immune response. Thus, the ability to study Goblet cell functions *in vitro* is an important feature of human colonoid cultures. One classical example for an association between mucus features and disease activity is found in ulcerative colitis where disease flares are associated with penetration of bacteria into the mucus layer and patients with active disease also present with a thinning layer (26, 27). Studies on Goblet cells in the context of pathophysiology and therapeutic intervention requires colonoid systems with epithelial cell differentiation that mimics as closely as possible what is observed *in vivo*. Our study demonstrates the high level of Goblet cells present in the mid colon *in vivo* in mice, which is recapitulated in our human and mouse colonoid systems. Detailing optimized conditions with regards to differentiation and provides a step forward for access to colonoids with a well-defined and differentiated Goblet cell compartment.

Mucosal epithelial cells play a crucial role in maintaining gut homeostasis and immunity not only by forming a protective barrier between luminal contents and the underlying mucosal immune system but also by secreting inflammatory mediators and signaling molecules that can influence local immune responses (7). Organoid models therefore offer an *in vitro* opportunity to study how gut epithelial cells can shape the tissue response under normal and pathological circumstances. In particular, we have shown that human colonoids represent a novel *in vitro* model of necroptosis that captures the characteristic features of cell death and the activation of immune-modulating inflammation pathways. Necroptosis is important for maintaining gut homeostasis under normal conditions, however when dysregulated it can also lead to the loss of barrier function and excessive release of DAMPs and other pro-inflammatory mediators that recruit and activate immune cells. which represent key events in the pathogenesis of intestinal inflammatory diseases such as IBD. Our data provide evidence that colonoids are a relevant model to study the molecular basis of necroptosis. Further development of this *in vitro* model system may provide valuable insights into novel therapeutic options for patients with diseases related to mucosal epithelial barrier dysfunction.

Given that adult stem cell-derived organoid cultures have been successfully established from several epithelial tissues, it would be interesting to determine whether we could expand our knowledge gained from colonoids to the culture of organoids from other epithelial tissues. However, even if all epithelial organoids rely on R-spondin and a BMP inhibitor such as Noggin or Gremlin 1 for their growth, they also require tissue-specific factors that mimic the tissue microenvironment of the stem cells to support their growth and differentiation. It is therefore unlikely that the methods described here with colonoids will be applicable to organoids from other epithelial tissues. Future work will therefore be critical to optimize the culture conditions for growing other organoids.

If the colonoid culture conditions as established in our study will maintain disease- specific features of epithelial cell alterations, is an important next question (28). Assuming that certain pathologic features are carried by the adult stem cell compartment, this is a likely scenario. Alternatively, the optimized culture conditions might overcome disease specific characteristics, if the penetration of the functional impairment is not complete and optimized culture gives unaffected stem cells a growth advantage. Such a situation might be comparable to overcoming the patient-dependent proliferation effects that were lost in the Wnt3a-conditioned medium as observed in our experiments. Reports in the literature, however, imply that disease-specific pathophysiological characteristics are maintained in intestinal organoid cultures (29). Before this important question can be addressed further, a more detailed resolution of the composition of the intestinal epithelium in different diseases and disease stages might be important (30). Once generation of additional comprehensive cellular atlases of the epithelial barrier in health and disease has progressed, reference points for a better characterization of *in vitro* cultures will be available (17, 31)

In summary, the culture strategies as detailed in the current study translate to the development of a standardized culture platform for *in vitro* models derived from mouse and human colon tissue. While science critically appreciates the fact that all organoid models face limitations (32), establishing conditions that allow researchers to generate reproducible culture systems will facilitate interpretation of outcomes across sites (33). As such, this study provides a rational framework for the development of colonoid cultures that more faithfully recapitulate the three-dimensional microenvironment of the epithelial barrier and, therefore, makes progress towards a system that models the intestinal microenvironment of human tissues *in vivo* for functional studies *in vitro*. Going forward, the availability of well-characterized colonoid culture conditions is anticipated to rapidly progress the development of additional platforms for preclinical evaluation of therapeutic strategies that improved epithelial barrier functions in disease.

## Data Availability Statement

The raw data supporting the conclusions of this article will be made available by the authors, without undue reservation.

## Ethics Statement

The studies involving human participants were reviewed and approved by Institutional Review Board of University of Massachusetts School of Medicine. The patients/participants provided their written informed consent to participate in this study. The animal study was reviewed and approved by Institutional Animal Care and Use Committee of AbbVie.

## Author Contributions

SWi, ST- study concept and design. SWi, MM, HK, TM, LPa, XW- acquisition of data. SWi, MM, TM, SWe, RD, ST, LPa, XW, LPh- analysis and interpretation of data. SWi, ST, EF- drafting of the manuscript. All- critical revision of the manuscript for important intellectual contributions. All authors contributed to the article and approved the submitted version.

## Funding

All authors are employees of AbbVie and financial support for this research were provided by AbbVie.

## Conflict of Interest

All authors are employees of AbbVie. The design, study conduct, and financial support for this research were provided by AbbVie. AbbVie participated in the interpretation of data, review, and approval of the publication.

## References

[1] Klunder LJ Faber KN Dijkstra G van ISCD. Mechanisms of Cell Polarity-Controlled Epithelial Homeostasis and Immunity in the Intestine. Cold Spring Harb Perspect Biol (2016) 9:a027888. 10.1101/cshperspect.a027888 PMC5495056 28213466

[2] Ananthakrishnan AN Bernstein CN Iliopoulos D Macpherson A Neurath MF Ali RAR. Environmental triggers in IBD: a review of progress and evidence. Nat Rev Gastroenterol Hepatol (2018) 15(1):39–49. 10.1038/nrgastro.2017.136 29018271

[3] Kaplan GG. The global burden of IBD: from 2015 to 2025. Nat Rev Gastroenterol Hepatol (2015) 12(12):720–7. 10.1038/nrgastro.2015.150 26323879

[4] Tordesillas L Berin MC Sampson HA. Immunology of Food Allergy. Immunity (2017) 47(1):32–50. 10.1016/j.immuni.2017.07.004 28723552

[5] Roda G Sartini A Zambon E Calafiore A Marocchi M Caponi A. Intestinal epithelial cells in inflammatory bowel diseases. World J Gastroenterol (2010) 16(34):4264–71. 10.3748/wjg.v16.i34.4264 PMC2937106 20818809

[6] Peterson LW Artis D. Intestinal epithelial cells: regulators of barrier function and immune homeostasis. Nat Rev Immunol (2014) 14(3):141–53. 10.1038/nri3608 24566914

[7] France MM Turner JR. The mucosal barrier at a glance. J Cell Sci (2017) 130(2):307–14. 10.1242/jcs.193482 PMC5278669 28062847

[8] Lancaster MA Knoblich JA. Organogenesis in a dish: modeling development and disease using organoid technologies. Science (2014) 345(6194):1247125. 10.1126/science.1247125 25035496

[9] Blutt SE Crawford SE Ramani S Zou WY Estes MK. Engineered Human Gastrointestinal Cultures to Study the Microbiome and Infectious Diseases. Cell Mol Gastroenterol Hepatol (2018) 5(3):241–51. 10.1016/j.jcmgh.2017.12.001 PMC5904028 29675450

[10] Sato T Vries RG Snippert HJ van de Wetering M Barker N Stange DE. Single Lgr5 stem cells build crypt-villus structures in vitro without a mesenchymal niche. Nature (2009) 459(7244):262–5. 10.1038/nature07935 19329995

[11] Sato T Stange DE Ferrante M Vries RG Van Es JH Van den Brink S. Long-term expansion of epithelial organoids from human colon, adenoma, adenocarcinoma, and Barrett’s epithelium. Gastroenterology (2011) 141(5):1762–72. 10.1053/j.gastro.2011.07.050 21889923

[12] Spence JR Mayhew CN Rankin SA Kuhar MF Vallance JE Tolle K. Directed differentiation of human pluripotent stem cells into intestinal tissue in vitro. Nature (2011) 470(7332):105–9. 10.1038/nature09691 PMC3033971 21151107

[13] Sato T Clevers H. Growing self-organizing mini-guts from a single intestinal stem cell: mechanism and applications. Science (2013) 340(6137):1190–4. 10.1126/science.1234852 23744940

[14] VanDussen KL Marinshaw JM Shaikh N Miyoshi H Moon C Tarr PI. Development of an enhanced human gastrointestinal epithelial culture system to facilitate patient-based assays. Gut (2015) 64(6):911–20. 10.1136/gutjnl-2013-306651 PMC4305344 25007816

[15] Ootani A Li X Sangiorgi E Ho QT Ueno H Toda S. Sustained in vitro intestinal epithelial culture within a Wnt-dependent stem cell niche. Nat Med (2009) 15(6):701–6. 10.1038/nm.1951 PMC2919216 19398967

[16] VanDussen KL Sonnek NM Stappenbeck TS. L-WRN conditioned medium for gastrointestinal epithelial stem cell culture shows replicable batch-to-batch activity levels across multiple research teams. Stem Cell Res (2019) 37:101430. 10.1016/j.scr.2019.101430 30933720 PMC6579736

[17] Haber AL Biton M Rogel N Herbst RH Shekhar K Smillie C. A single-cell survey of the small intestinal epithelium. Nature (2017) 551(7680):333–9. 10.1038/nature24489 PMC6022292 29144463

[18] Tanay A Regev A. Scaling single-cell genomics from phenomenology to mechanism. Nature (2017) 541(7637):331–8. 10.1038/nature21350 PMC5438464 28102262

[19] Wilson SS Tocchi A Holly MK Parks WC Smith JG. A small intestinal organoid model of non-invasive enteric pathogen-epithelial cell interactions. Mucosal Immunol (2015) 8(2):352–61. 10.1038/mi.2014.72 PMC4326599 25118165

[20] Miyoshi H Stappenbeck TS. In vitro expansion and genetic modification of gastrointestinal stem cells in spheroid culture. Nat Protoc (2013) 8(12):2471–82. 10.1038/nprot.2013.153 PMC3969856 24232249

[21] Watanabe K Ueno M Kamiya D Nishiyama A Matsumura M Wataya T. A ROCK inhibitor permits survival of dissociated human embryonic stem cells. Nat Biotechnol (2007) 25(6):681–6. 10.1038/nbt1310 17529971

[22] van Putten JPM Strijbis K. Transmembrane Mucins: Signaling Receptors at the Intersection of Inflammation and Cancer. J Innate Immun (2017) 9(3):281–99. 10.1159/000453594 PMC5516414 28052300

[23] Pasparakis M Vandenabeele P. Necroptosis and its role in inflammation. Nature (2015) 517(7534):311–20. 10.1038/nature14191 25592536

[24] Birchenough GM Johansson ME Gustafsson JK Bergstrom JH Hansson GC. New developments in goblet cell mucus secretion and function. Mucosal Immunol (2015) 8(4):712–9. 10.1038/mi.2015.32 PMC4631840 25872481

[25] Pelaseyed T Bergstrom JH Gustafsson JK Ermund A Birchenough GM Schutte A. The mucus and mucins of the goblet cells and enterocytes provide the first defense line of the gastrointestinal tract and interact with the immune system. Immunol Rev (2014) 260(1):8–20. 10.1111/imr.12182 24942678 PMC4281373

[26] Johansson ME Hansson GC. Immunological aspects of intestinal mucus and mucins. Nat Rev Immunol (2016) 16(10):639–49. 10.1038/nri.2016.88 PMC6435297 27498766

[27] Pullan RD Thomas GA Rhodes M Newcombe RG Williams GT Allen A. Thickness of adherent mucus gel on colonic mucosa in humans and its relevance to colitis. Gut (1994) 35(3):353–9. 10.1136/gut.35.3.353 PMC1374589 8150346

[28] Mead BE Ordovas-Montanes J Braun AP Levy LE Bhargava P Szucs MJ. Harnessing single-cell genomics to improve the physiological fidelity of organoid-derived cell types. BMC Biol (2018) 16(1):62. 10.1186/s12915-018-0527-2 29871632 PMC5989470

[29] Freire R Ingano L Serena G Cetinbas M Anselmo A Sapone A. Human gut derived-organoids provide model to study gluten response and effects of microbiota-derived molecules in celiac disease. Sci Rep (2019) 9(1):7029. 10.1038/s41598-019-43426-w 31065051 PMC6505524

[30] Kinchen J Chen HH Parikh K Antanaviciute A Jagielowicz M Fawkner-Corbett D. Structural Remodeling of the Human Colonic Mesenchyme in Inflammatory Bowel Disease. Cell (2018) 175(2):372–86.e17. 10.1016/j.cell.2018.08.067 30270042 PMC6176871

[31] Regev A Teichmann SA Lander ES Amit I Benoist C Birney E. The Human Cell Atlas. Elife (2017) 6:e27041. 10.7554/eLife.27041 29206104 PMC5762154

[32] Mead BE Karp JM. All models are wrong, but some organoids may be useful. Genome Biol (2019) 20(1):66. 10.1186/s13059-019-1677-4 30917855 PMC6437983

[33] Bar-Ephraim YE Kretzschmar K Clevers H. Organoids in immunological research. Nat Rev Immunol (2020) 20(5):279–93. 10.1038/s41577-019-0248-y 31853049

