# Supplementary material for: Optimized Culture Conditions for Improved Growth and Functional Differentiation of Mouse and Human Colon Organoids
Source: Front Immunol. 2021 Feb 12;11:547102. doi: 10.3389/fimmu.2020.547102 (PMC7906999; doi:10.3389/fimmu.2020.547102)

Supplementary Material

**Supplementary Figure 1.** **A direct correlation exists between luminescence measured with the Cell Titer-Glo 3D assay and the number of colonoids in culture.** (A) Mouse and (B) human colonoids were cultured in WNR CM medium for three days and then assayed by Cell Titer-Glo 3D and counting of the number of organoids per visual field. In each experiment at each dilution z-stacks of 3 wells were acquired at intervals of 99 microns to cover the entire Matrigel plug using a 2x and 4x objective on a Keyence BZ-X800 microscope. Full focus images were obtained from the z-stacks and every organoid per image counted using the cell counter Plugin from ImageJ. For Cell-Titer Glo 3D enumeration, media was aspirated from the 3 wells and the Matrigel plug was incubated in and equal volume of BD cell recovery solution and Cell-titer Glo 3D before the solution was transferred to a 96 well black plate and luminescence read on a Biotek Synergy 2. In A and B, representative 2x and 4x bright field images are shown at 2-fold dilutions of cells. Scale bars indicate 100 microns and error bars represent SD from two independent experiments.

**Supplementary Figure 2. Recombinant Wnt3a does not support maintenance and propagation of stem cells in mouse colonoids.**. (A) Representative images of mouse colonoids grown for six days in WNR CM (left two panels) or for two days in WNR CM followed by 4 days in rENR media (right two panels). 4x brightfield and 20x IHC images are shown. Mouse colonoids derived from C57Bl/6 mice crypts were cultured for two days in WRN CM, Wnt3a CM + rENR or rWENR medium before medium change to rENR or maintenance in original media. After four additional days of culture, colonoids were assayed for markers of epithelial cell types by qPCR (B) Analysis of LGR5 transcript levels; (C) Analysis of Axin2 transcript levels; (D) Analysis of Mucin 2 transcript levels; (E) Analysis of ChgA transcript levels for monitoring enteroendocrine cells. Scale bars indicate 50 microns and error bars are representative of the average of biological triplicates from three independent experiments. Statistical significance was determined by one-way ANOVA.


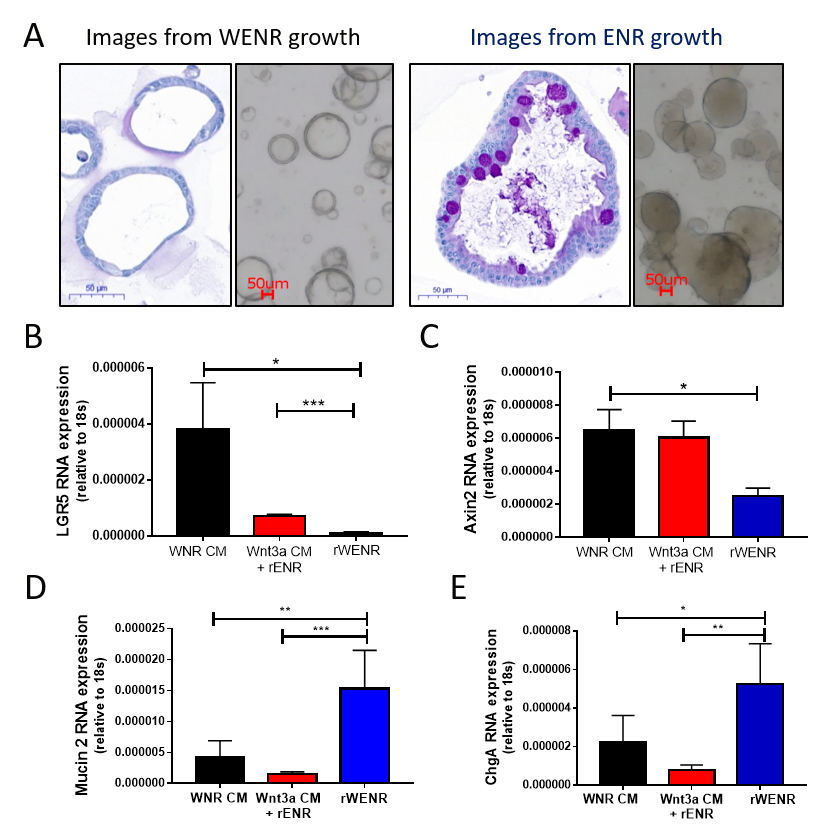

Supplement: Supplementary file 1 [file DataSheet_1.docx]
